# Mycobacterial Nucleoside Diphosphate Kinase Blocks Phagosome Maturation in Murine Raw 264.7 Macrophages

**DOI:** 10.1371/journal.pone.0008769

**Published:** 2010-01-19

**Authors:** Jim Sun, Xuetao Wang, Alice Lau, Ting-Yu Angela Liao, Cecilia Bucci, Zakaria Hmama

**Affiliations:** 1 Division of Infectious Diseases, Department of Medicine, University of British Columbia and Vancouver Coastal Health Research Institute, Vancouver, British Columbia, Canada; 2 Department of Biological and Environmental Sciences and Technologies, University of Salento, Lecce, Italy; University of Delhi, India

## Abstract

**Background:**

Microorganisms capable of surviving within macrophages are rare, but represent very successful pathogens. One of them is *Mycobacterium tuberculosis* (*Mtb*) whose resistance to early mechanisms of macrophage killing and failure of its phagosomes to fuse with lysosomes causes tuberculosis (TB) disease in humans. Thus, defining the mechanisms of phagosome maturation arrest and identifying mycobacterial factors responsible for it are key to rational design of novel drugs for the treatment of TB. Previous studies have shown that *Mtb* and the related vaccine strain, *M. bovis* bacille Calmette-Guérin (BCG), disrupt the normal function of host Rab5 and Rab7, two small GTPases that are instrumental in the control of phagosome fusion with early endosomes and late endosomes/lysosomes respectively.

**Methodology/Principal Findings:**

Here we show that recombinant *Mtb* nucleoside diphosphate kinase (Ndk) exhibits GTPase activating protein (GAP) activity towards Rab5 and Rab7. Then, using a model of latex bead phagosomes, we demonstrated that Ndk inhibits phagosome maturation and fusion with lysosomes in murine RAW 264.7 macrophages. Maturation arrest of phagosomes containing Ndk-beads was associated with the inactivation of both Rab5 and Rab7 as evidenced by the lack of recruitment of their respective effectors EEA1 (early endosome antigen 1) and RILP (Rab7-interacting lysosomal protein). Consistent with these findings, macrophage infection with an Ndk knocked-down BCG strain resulted in increased fusion of its phagosome with lysosomes along with decreased survival of the mutant.

**Conclusion:**

Our findings provide evidence in support of the hypothesis that mycobacterial Ndk is a putative virulence factor that inhibits phagosome maturation and promotes survival of mycobacteria within the macrophage.

## Introduction

Tuberculosis (TB) is a devastating disease caused by *Mycobacterium tuberculosis* (*Mtb*), which claims about 2 million lives every year [1]. Moreover, the emergence of drug resistant *Mtb* strains and their spread to the general population now pose unprecedented difficulties to the control of TB disease [2]. Given the persistent global burden of TB, it is crucial that research delineate the underlying mechanisms of *Mtb* pathogenesis in order to pave the road for developing better strategies to prevent and treat TB.

The ability of *Mtb* to persist and replicate within the host macrophage is a central factor in the development of TB disease [3]. Intracellular survival of *Mtb* is aided by a combination of factors including a unique cell wall structure, which physically shields the bacterium from bactericidal and hydrolytic enzymes [4], and secretion of enzymes to combat host reactive oxygen and nitrogen radicals [5], [6]. Although all these factors contribute to *Mtb* persistence within the macrophage, one recurring and highly important feature of this pathogen is inhibition of normal phagosome maturation process, thereby abrogating physical fusion of phagosome with lysosomes and ultimately protecting the bacterium from a bactericidal environment [7], [8], [9].

Phagosome biogenesis is characterized by a rapid and sequential fusion of vacuoles containing ingested pathogens with various endosomal compartments leading to acidification dependent on recruitment of the vacuolar proton ATPase subunits [8]. Thereafter, the acquisition of acidic lysosomal enzymes by the phagosome and their activation results in efficient killing and degradation of invading pathogens [10] from which the macrophage switch to the function of antigen presentation for proper detection by effectors of the adaptive immune response [10], [11].

Rab GTPases play a major role in the control of normal phagosome biogenesis. Normally, phagosome biogenesis is initiated by fusion with endosomes coated with the small GTPase, Rab5. This step is essential for recruitment of the early endosome antigen 1 (EEA1), which drives the phagosome towards further maturation [12]. However, this early maturation event is disrupted by *Mtb* and the closely related vaccine strain *M. bovis* BCG, both of which exclude EEA1 from their phagosomes [13]. As the phagosome matures into more advanced stages, another prominent member of late phagosome markers, the GTPase Rab7 is recruited and serves as a docking site for RILP (Rab7-interacting lysosomal protein). RILP possesses two distinct domains: one that binds to the GTP-bound form of Rab7 and another that recruits the dynein/dynactin complex [14], [15]. By simultaneously associating with both targets, RILP promotes the interaction of vesicles bearing active Rab7 with lysosomes [14]. Initially, one group demonstrated that *Mtb* phagosomes retained Rab7 on the phagosome despite arresting its maturation to phagolysosomes [16]. Our recent studies have furthered advanced these findings and demonstrated that Rab7 molecules on the membrane of mycobacterial phagosome are inactivated by secreted factor(s) from live pathogenic mycobacteria [17]. Therefore, mycobacteria disrupt phagolysosome fusion in a mechanism dependent on Rab7-RILP interaction.

Rab cycling is a unique and essential characteristic of small GTPases including Rab5 and Rab7. These proteins bind GDP/GTP and to be functionally active, they must be in the GTP-bound state. Our previous studies demonstrated that mycobacteria interfere with this cycling through a GTPase activating protein (GAP)-like activity, which depletes the γ-phosphate from GTP-bound Rab7 molecules [17].

The finding that live mycobacteria export a variety of proteins and glycolipids intracellularly [18], [19], [20], [21] and the demonstration that proteins with subunit size up to 70 kDa are able to cross the phagosomal membrane towards the cytosol supported the search for secreted mycobacterial products that might interact with and inhibit critical regulators of phagosome biogenesis [22], [23]. In this context, our search for a secreted mycobacterial factor that would interfere with Rab7 activation identified nucleoside diphosphate kinase (Ndk) as a prominent candidate. Ndk is a ubiquitous small protein (∼15 kDa) found in virtually all organisms, from eukaryotes to prokaryotes [24], [25]. Initially, mycobacterial Ndk was described as an intracellular nucleotide pool balance mediator [25] because it has several enzymatic properties such as autophosphorylation and GTPase activity, as well as phosphotransfer activities [26], [27]. More importantly Ndk is now known to be secreted by mycobacteria, including *Mtb* and BCG [28], [29] and *in vitro* analyses have demonstrated that *Mtb* Ndk possesses GAP activity towards Rho GTPases [26]. This raises the question as to whether mycobacterial Ndk also alters the function of Rab GTPases, which has implications for phagosome maturation arrest and survival within the host macrophage.

Here, we show that recombinant *Mtb* Ndk dephosphorylates Rab7-GTP and also Rab5-GTP in a cell-free biochemical assay. In contrast, recombinant Ndk from *M. smegmatis*, a non-pathogenic mycobacteria, had little or no effect on Rab molecules. Furthermore, we show that phagosomes containing latex beads coated with *Mtb* Ndk resist fusion with lysosomes, consistent with the finding that phagosomes containing a BCG strain with knocked-down Ndk matures at higher rate leading ultimately to increased intracellular killing.

## Results

### Ndk from Pathogenic Mycobacteria Deactivates Rab5 and Rab7 GTPases

**Figure 1:**
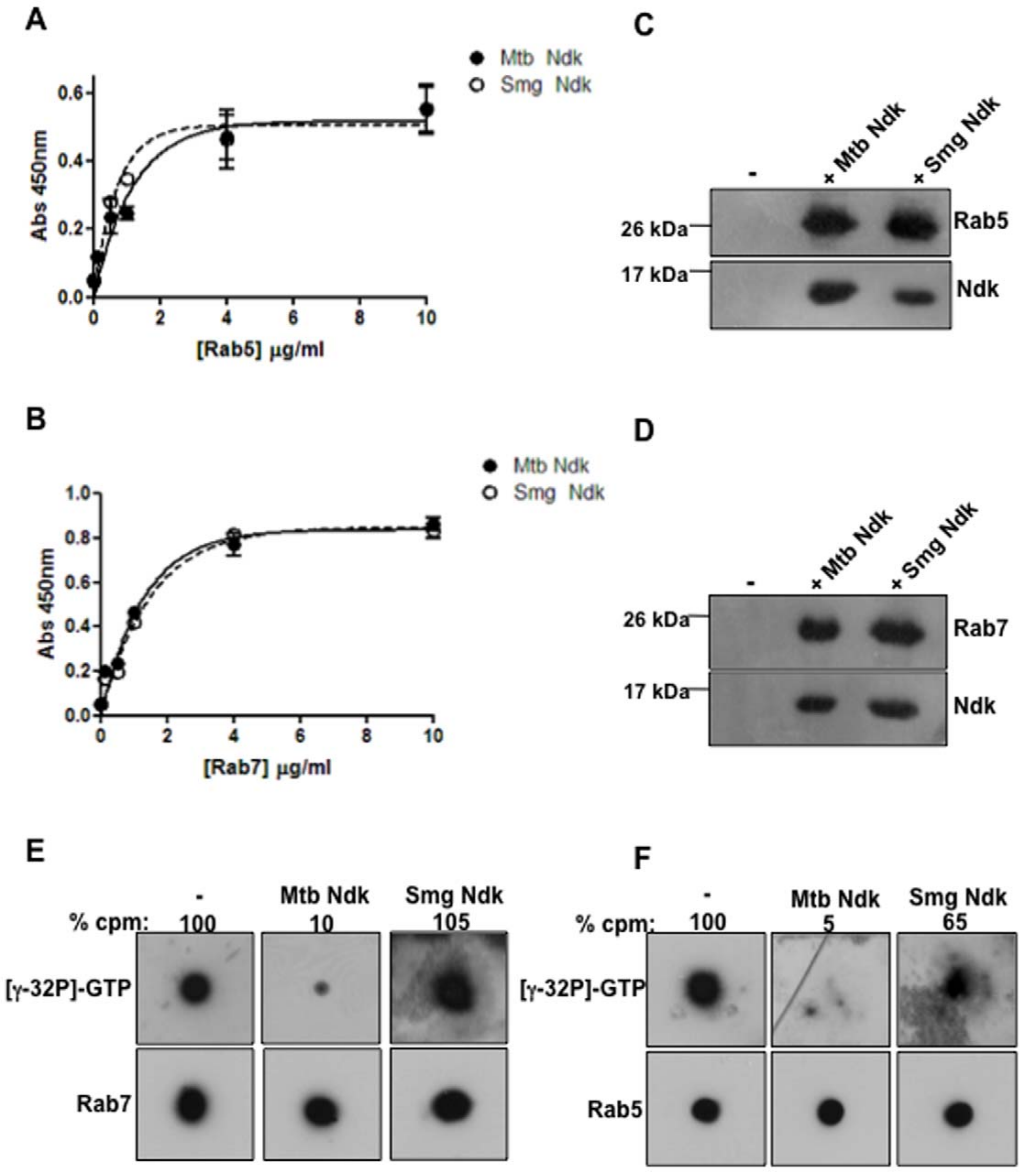
Ndk interacts with and deactivates Rab5 and Rab7 GTPases. A and B. ELISA microplates were coated with 10 µg/ml *Mtb* or *M. smegmatis* Ndk or control BSA, and incubated for 1 h with increasing concentrations of Rab5 or Rab7, previously loaded with 1 mM GTP in reaction buffer (50 mM HEPES pH 7.4, 50 mM NaCl, 0.1 mM DTT, 5 mM EDTA and 1 mg/ml BSA) for 10 min at 37°C. Following 3 washes, attached Rab5 or Rab7 was probed with primary rabbit anti-Rab5 or Rab7, followed by secondary anti-rabbit-HRP conjugate. Thereafter, the interaction was visualized at absorbance 450 nm after addition of TMB substrate. Values from control BSA were subtracted. Results (mean ± s.e.m) are from 3 independent experiments. **C and D.** Recombinant Ndk (3 µg) and GTP-loaded Rab5 or Rab7 (3 µg) were incubated in PBS buffer for 1 h at 4°C. Thereafter, anti-Ndk antibodies (1∶100) were added (1 h at 4°C) and subjected to immunoprecipitation with protein A agarose beads for 30 min at room temperature. Samples were washed three times then analyzed by SDS-PAGE and western blot with anti-Ndk and anti-Rab5 or anti-Rab7 followed subsequently by monoclonal anti-rabbit IgG, native-peroxidase. Pulled down Rab7 and Rab5 are shown as the 25 kDa and 27 kDa protein bands respectively, while the 15 kDa protein band correspond to Ndk. **E and F.** Recombinant Rab5 and Rab7 were loaded with [γ-^32^P]-GTP and spotted onto nitrocellulose membranes. After extensive washes, membranes were either left untreated or incubated with recombinant *Mtb* or *M. smegmatis* Ndk at room temperature for 2 h. Membranes were washed, dried and exposed to X-ray film (upper panel). The radioactive signal observed depicts remaining active GTP-Rab7 or -Rab5 on membranes and values are quantification of bound [γ-^32^P]-GTP relative to control untreated samples as determined by radioactive count in a liquid scintillation counter. After film development, membranes were probed with anti-Rab7 to ensure equal spotting of Rab7 protein (lower panel).

Based upon a previously published work showing that secreted *Mtb* Ndk manipulates the Rho GTPase regulatory cycle [26] and our recent observation that pathogenic mycobacteria express GAP activity towards Rab7 GTPase [17], we over-expressed and purified recombinant *Mtb* Ndk to homogeneity and examined its interaction with both Rab7, and the closely-related molecule, Rab5. Recombinant Ndk from the non-pathogenic *M. smegmatis* was also included in these experiments. We first examined Ndk binding to Rab molecules. Recombinant *Mtb* or *M. smegmatis* Ndk were coated onto flat bottom 96 well plates and exposed to increasing concentrations of Rab5 or Rab7. As illustrated in Figure 1, both *M smegmatis* and *Mtb* Ndk bind to Rab5 (**Fig. 1A**) and Rab7 (**Fig. 1B**) in a dose-dependent manner. No difference in binding characteristics was observed between *M. smegmatis* and *Mtb* Ndk. In other experiments, soluble Rab7 and Rab5 were incubated with Ndk then subjected to immunoprecipitation with anti-Ndk antibodies and protein A agarose beads. SDS-PAGE and western blotting analysis of pulled-down material with specific antibodies showed that Ndk interacts effectively with both Rab5 and Rab7 GTPases (**Fig. 1C and 1D**), thus confirming the solid phase binding assays. We next examined whether bound Ndk expresses GAP activity towards Rab molecules. Rab5 and Rab7 proteins were preloaded with radioactive GTP as described in the Material and Method section. GTP-bound Rab proteins were then spotted onto nitrocellulose membrane squares and incubated in presence and absence of *Mtb* or *M. smegmatis* Ndk and washed. After exposure to X-ray films, membranes were probed with either anti-Rab5 or anti-Rab7 antibodies. The blotting analysis confirmed equal loading of Rab5 and Rab7 proteins on membrane squares (**Fig. 1E** and **1F**, lower panels) and the radioactive signal (**Fig. 1E** and **1F**, top panels) clearly demonstrated that *Mtb* Ndk is able to dephosphorylate the γ-phosphate of bound GTP to Rab5 and Rab7. Quantification of the radioactivity showed 95% and 90% depletion of gamma phosphate from both Rab5 and Rab7, respectively. In contrast, *M. smegmatis* Ndk dephosphorylated Rab5 only partially (35% reduction of GTP) (**Fig. 1F**) and had almost no effect on Rab7 (**Fig. 1E**) despite efficient binding to both GTPases (**Fig. 1A** and **1B**). These findings demonstrated that Ndk from pathogenic mycobacteria expresses strong GAP activity towards Rab5 and Rab7 GTPases.

### Ndk from Pathogenic Mycobacteria Inhibits Phagolysosome Fusion

**Figure 2:**
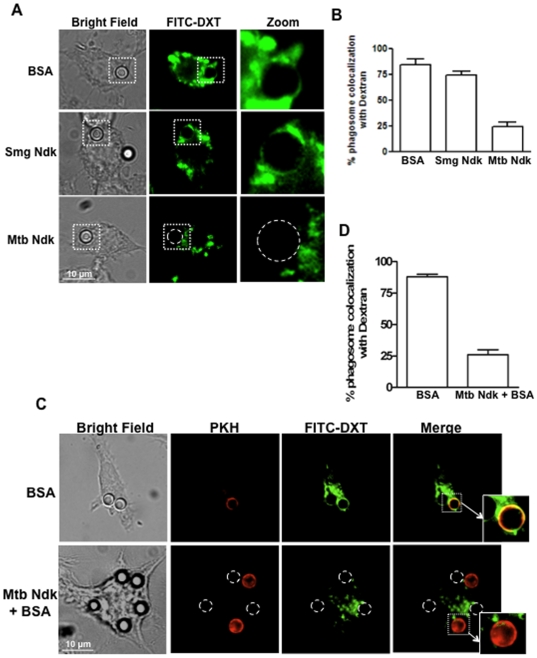
Ndk contributes to phagosome maturation arrest. **A.** RAW 264.7 cells were pulse-chased overnight with FITC-DXT (0.5 mg/ml) and allowed to ingest control BSA-coated, *Mtb* Ndk-coated, or *M. smegmatis* Ndk-coated latex beads. Two hours post-phagocytosis, cells were washed and fixed for analysis by confocal microscopy. **B.** Quantification of the confocal data shown in panel A. **C.** RAW 264.7 cells were loaded with FITC-DXT overnight and allowed to ingest a mixture (1∶1) of either PKH-labelled BSA-beads and unlabelled BSA-beads (upper panel, control) or PKH-labelled BSA-beads and unlabelled Ndk-beads (lower panel). Two hours post-phagocytosis, cells were trypsinized, washed, and fixed for analysis by confocal microscopy. The top panel (BSA control), the yellow signal reflects a colocalization of the PKH-BSA-beads with the lysosomal marker dextran. In the bottom panel dotted circles indicate the position of Ndk-beads, while the red fluorescent signal (PKH) shows the location of BSA-beads and a significant decrease of dextran colocalization with distant PKH-BSA-beads. **D.** Quantification of the confocal data shown in panel C. Values in B and D are the mean ± SD of phagosome colocalization of with FITC-DXT in 50–80 cells from three independent experiments.

Rab5- and Rab7-regulated endosomal trafficking in macrophages is known to be dependent on GTP binding [30], [31]. Therefore, the *in vitro* data showing that Rab5-GTP and Rab7-GTP are potential substrates for Ndk GAP activity (**Fig. 1**) suggested that Ndk might disrupt maturation of phagosome-containing pathogenic mycobacteria. To verify this hypothesis, we used the latex bead model for protein and glycolipid delivery into phagosomes [13], [23], [32] and examined the effect of Ndk on phagosome-lysosome fusion in RAW 264.7 macrophages. Thus, 4 µm latex beads were coated with *Mtb* or *M. smegmatis* Ndk or BSA (control). The efficiency of coating was regularly examined by SDS-PAGE and Coomassie Blue staining, and FACS analyses of beads labelled with specific antibodies (Data not shown). RAW cells were first pulse-chased with FITC-labelled dextran (FITC-DXT) and exposed to coated beads for 20 min at 4°C. Synchronous uptake was induced by temperature shift to 37°C and cells were incubated for 2 hr to allow for phagosome-lysosome fusion to occur. Cells were then fixed and examined by digital confocal microscopy. DXT is a non-biodegradable compound that accumulates in the lysosome and is commonly used to directly visualize fusion of phagosomes with lysosomes [33], [34]. The results illustrated in **Fig. 2A** and **2B** show that most vacuoles containing BSA-coated beads were uniformly surrounded with green-fluorescent vesicles indicative of fusion with lysosomes. In contrast, the images clearly demonstrated that phagosomes containing *Mtb* Ndk-coated beads did not reach lysosomes. Consistent with these findings, *M. smegmatis* Ndk, which has only minor effect on Rab GTPases, did not oppose bead phagosome fusion with lysosomes.

To examine whether Ndk dissociates from beads and exit the phagosomal membrane toward the cytosol to inhibit fusion with lysosomes, we analyzed FITC-DXT-loaded macrophages co-infected with red fluorescent (PKH-labelled) BSA-beads and unlabelled Ndk-beads (or BSA beads, control). The expectation was that protein released from Ndk-bead phagosomes would act on distant vacuoles containing red-fluorescent BSA-beads. Indeed, confocal images of cell co-infected with Ndk-beads and PKH-beads, (**Fig. 2C**, lower panel) and their quantification (**Fig. 2D**) showed that PKH-beads almost completely excluded FITC-DXT vesicles from their phagosomes as result of block of fusion with lysosomes. In contrast, an abundant green fluorescent signal surrounded PKH-phagosomes in cells co-infected with control beads coated with BSA (**Fig. 2C**, upper panel) indicative of fusion with DXT-loaded lysosomes. The observation of Ndk-mediated down-modulation of phagosome maturation cannot be attributed to a global toxicity of the host cell maturation. Indeed, the viability, morphology and adherence of RAW infected with Ndk-beads over 24 h culture period were similar to that of the control non-infected cells.

Collectively, these experiments suggest that secretory Ndk released from pathogenic mycobacteria within the phagosome might have access to the cytosolic face of the phagosomal membrane to interact with and inhibit effectors of phagosome maturation.

### *Mtb* Ndk Inhibits Recruitment of Rab5 Effectors to Phagosomes

**Figure 3:**
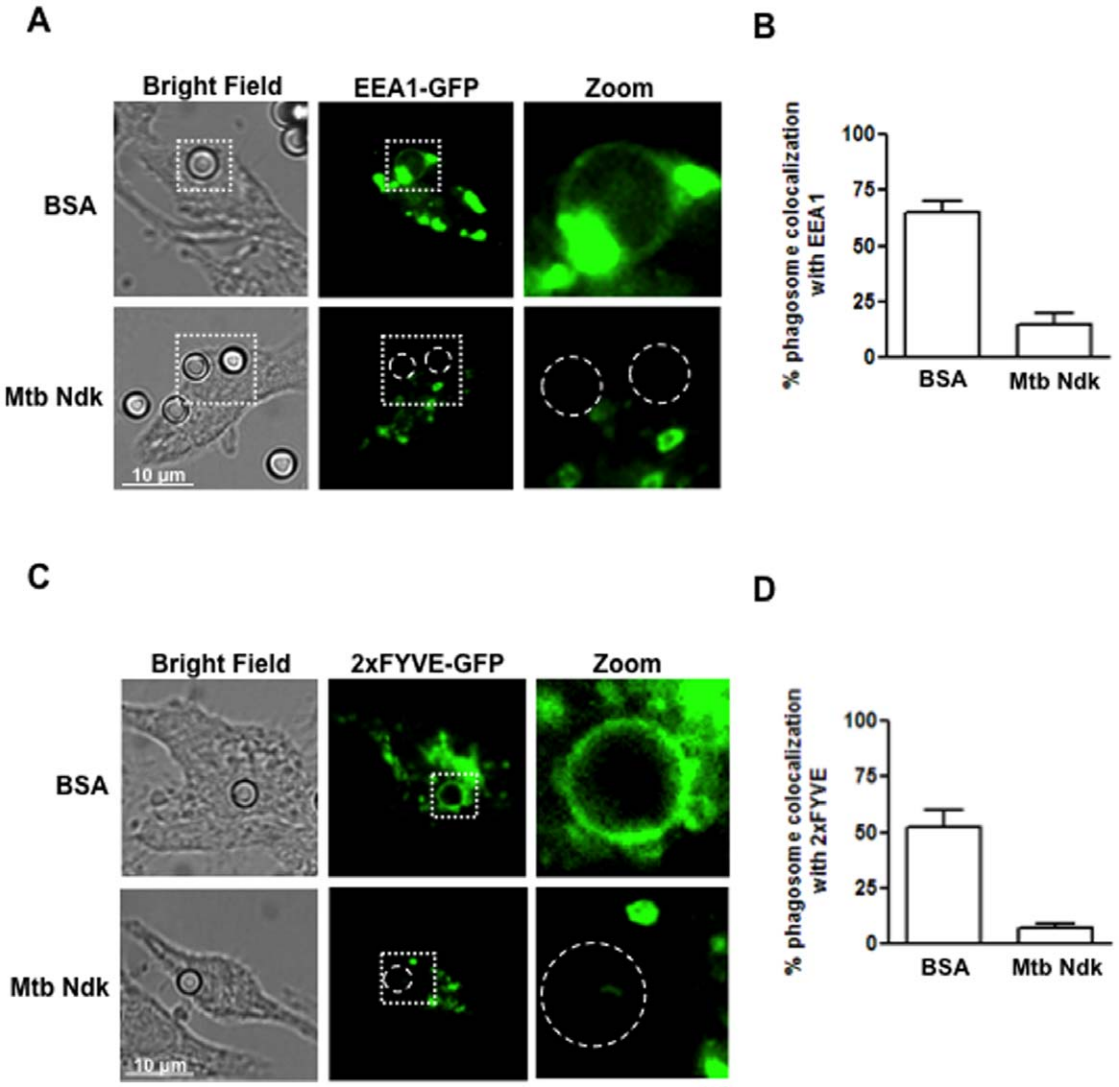
Ndk inhibits EEA1 recruitment to phagosomes. **A.** RAW cells were transfected with EEA1-GFP and thereafter allowed to ingest either control BSA- or Ndk-coated latex beads. The green signal shows presence of EEA1 on the phagosome containing control beads (upper panel), while the lack of signal around the phagosome (lower panel) shows diminished recruitment of EEA1 to Ndk-bead containing phagosomes. **B.** Quantification of the confocal data shown in panel A. **C.** Raw cells were transfected with 2xFYVE-GFP, and thereafter allowed to phagocytose either control BSA- or Ndk-coated latex beads. 2xFYVE is a specific marker for PI3P. The green signal seen in the upper control panel indicates an abundance of PI3P generated on the phagosome, while the lack of signal on Ndk-bead phagosomes indicate absence of PI3P. **D.** Quantification of confocal data shown in panel C. Values in B and D are the mean ± SD of phagosome colocalization with EEA1-GFP and 2xFYVE-GFP respectively in 50–80 cells from three independent experiments.

Membrane acquisition of EEA1 effector is an essential molecular event for phagosomal maturation [12]. Endosomal recruitment of EEA1 occurs via binding to active (GTP bound) Rab5 [30]. Given that pathogenic mycobacteria exclude EEA1 from their phagosomes [13] and that *Mtb* Ndk deactivates Rab5 (**Fig. 1F**) we examined whether Ndk interferes directly with the process of phagosomal recruitment of EEA1. To do so, we transiently transfected RAW macrophages with a chimera consisting of GFP fused to EEA1 then subjected them to phagocytosis of coated latex beads. Cells were examined by confocal microscopy 20 min after bead attachment to the cell membrane (**Fig. 3A**). In cells ingesting BSA-coated beads (control) about 65% bead phagosomes were surrounded by abundant green fluorescent signal reflecting normal recruitment of Rab5 effector EEA1 (**Fig. 3B**). In contrast, macrophages infected with Ndk-coated beads showed almost no recruitment of EEA1 to the phagosomes. EEA1 is recruited to endosomal membranes via binding of its FYVE domain to phosphatidylinositol 3-phosphate (PI3P), which results from phospahtidylinositol (PI) phosphorylation by the class III phosphoinositide 3-kinase enzyme hVPS34 [35]. Therefore we examined whether inhibition of EEA1 recruitment in the presence of Ndk is related to reduced PI3P formation on phagosomal membrane. RAW macrophages were transfected with a 2xFYVE-GFP construct, which is commonly used as a fluorescent probe for in situ detection of PI3P on endosomal membranes [35], [36]. Cells were then allowed to ingest coated latex beads and were examined by confocal microscopy. The images obtained (**Fig. 3C**) showed abundant recruitment of the FYVE domain to about 55% of phagosomes containing BSA-coated beads (**Fig. 3D**), reflecting a membrane enrichment in PI3P product, while most (95%) of Ndk-bead phagosomes excluded the fluorescent probe most likely due to a failure of PI phosphorylation by hVPS34. Given that hVPS34 binds to and is seemingly activated by GTP-bound (active) Rab5 [36], [37], our findings strongly suggest that *Mtb* Ndk interrupts hVPS34 recruitment to the phagosomes via dephosphorylation of Rab5-GTP.

### *Mtb* Ndk Inhibits Recruitment of RILP to Late Phagosomes

**Figure 4:**
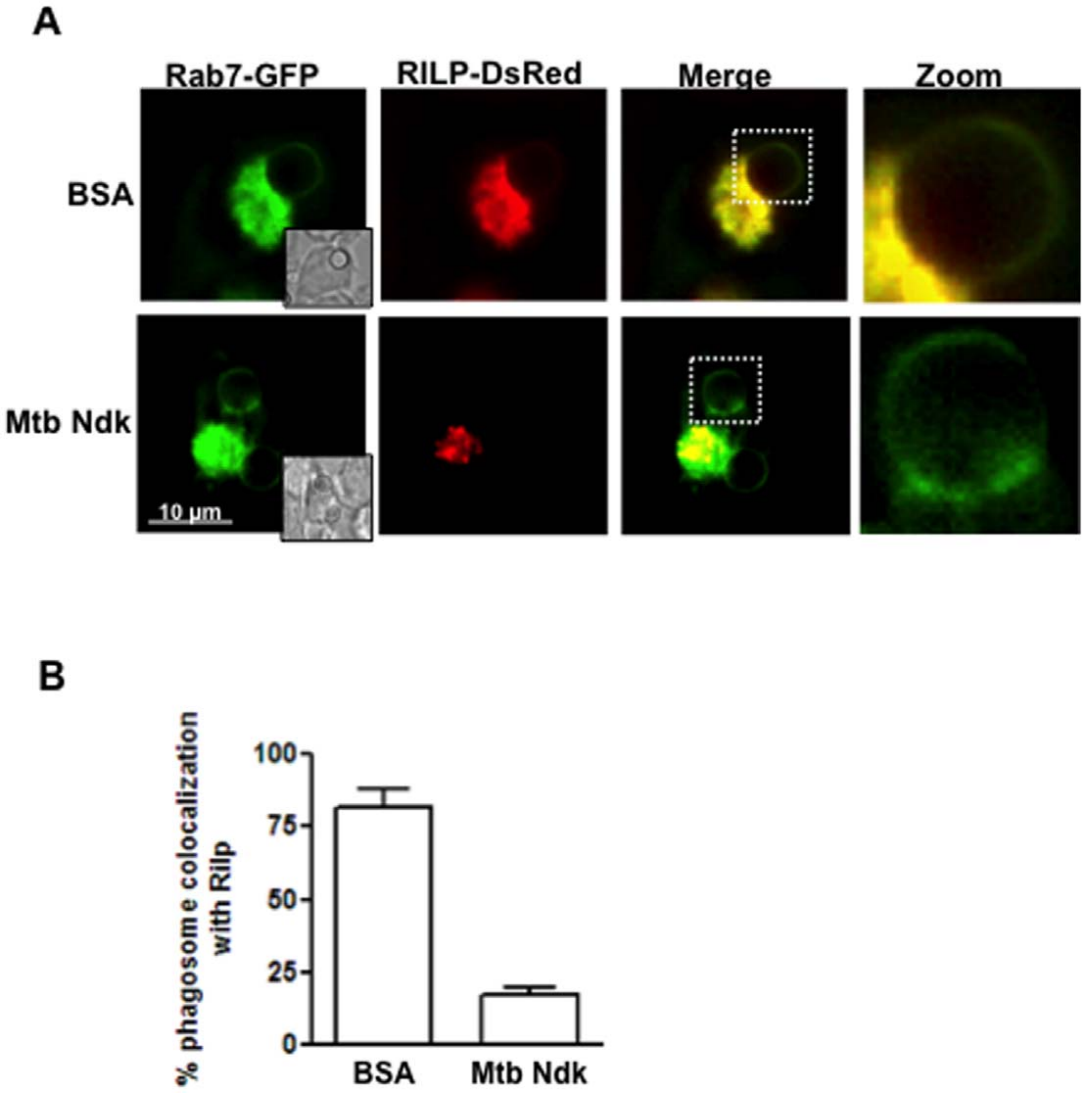
A. Ndk disrupt Rab7-RILP interaction. RAW cells were double transfected with Rab7-GFP and RILP-DsRed as described in Materials and Methods. Thereafter, cells were allowed to ingest either control BSA- or Ndk-coated latex beads. The yellow signal seen (upper panel) shows colocalization of Rab7 and RILP on the phagosome. The green signal (lower panel) shows phagosomes positive for Rab7 but no recruitment of RILP. **B.** Quantification of the confocal data shown in panel A. Values in B are the mean ± SD of phagosome colocalization with RILP-DsRed in 50–80 cells from three independent experiments.

Fusion of late phagosomes with lysosomes is dependent upon interaction of Rab7 molecules with effector molecules RILP [14]. We have recently demonstrated that macrophage infection with live BCG inhibited RILP recruitment despite acquisition of detectable amount of Rab7 on the phagosome. Given that phagosomal recruitment of RILP occurs via binding to active (GTP bound) Rab7 [38], [39] and the observation made here that Ndk catalyzes the GTP/GDP switch on recombinant Rab7 molecules (**Fig. 1**), it is likely that abortion of Rab7-RILP interaction in infected macrophages results from the export of Ndk by mycobacterium within the phagosome. To verify this prediction, we double transfected RAW macrophages with Rab7-GFP and RILP-DsRed and generated phagosomes with BSA- or Ndk-coated beads. The results obtained from confocal analyses (**Fig. 4A**) and their quantification (**Fig. 4B**) showed a strong colocalization of red and green signals on a large number (75%) of phagosomes containing BSA-beads, indicating normal recruitment of Rab7 and its effector molecule RILP. In contrast, most Ndk-bead-containing phagosomes (>80%) excluded RILP from their membranes despite a substantial acquisition of Rab7 molecules (**Fig. 4A**). These findings established a correlation between phagolysosome fusion arrest observed above (**Fig. 2A** and **2C**) and Ndk-dependent disruption of Rab7-RILP interaction.

### Antisense Inhibition of Ndk Expression Attenuates Survival of BCG in Macrophages

**Figure 5:**
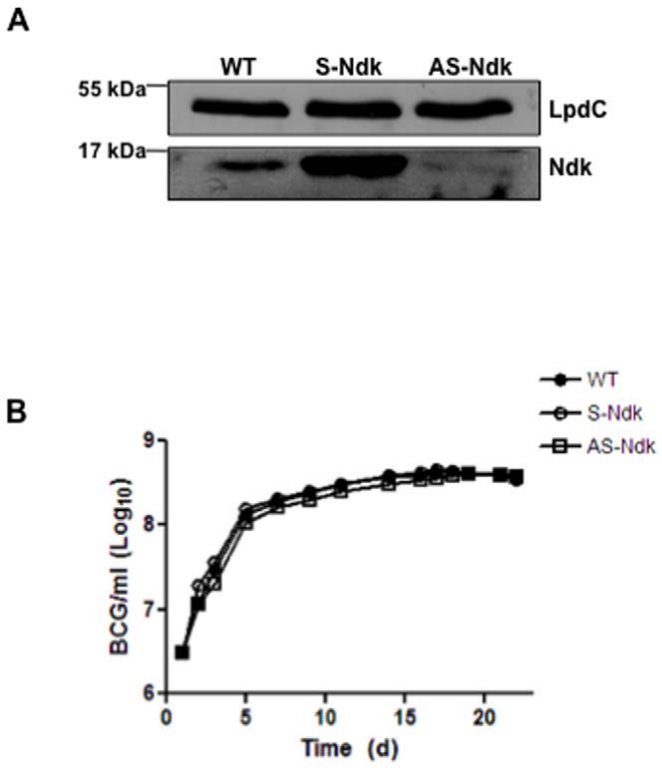
Generation of recombinant BCG with knocked down Ndk expression. **A.** Wild-type BCG, BCG transformed with pMV261-S-Ndk (sense, overexpression), and BCG transformed with pMV261-AS-Ndk (antisense, knockdown) were lysed as described in Materials and Methods, and subjected to 15% SDS-PAGE, followed by western blot with anti-Ndk antibodies. Mycobacterial lipoamide dehydrogenase C (LpdC) was used as an internal control for equal loading. **B.** Growth curve of the BCG strains shown in panel A expressed as Absorbance at 600 nm.

**Figure 6:**
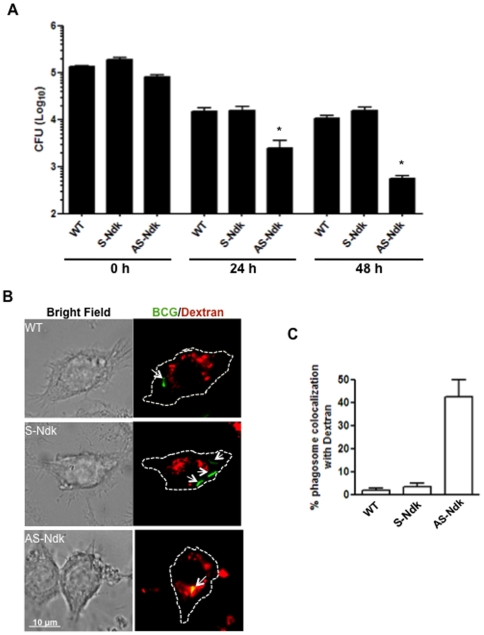
BCG-AS−Ndk has decreased intracellular survival due to increased phagolysosome fusion. **A.** RAW macrophages were infected with BCG strains (MOI of 10∶1). Then culture media was supplemented with 50 µg/ml gentamicin to kill extracellular non-ingested mycobacteria. Cells were washed three times in PBS and lysed in 0.025% SDS 1 h (0 h time point), 24 h, and 48 h post-infection. Serial dilutions of recovered bacteria were then plated on solid 7H10 media supplemented with 10% OADC. CFU counts were performed after 3 weeks incubation at 37°C,. Bars, mean ± s.e.m. (three independent experiments). **B.** RAW cells adherent to cover slips were loaded with 0.5 mg/ml Texas Red-Dextran overnight and then infected with FITC-labelled BCG strains (wild-type: WT, expression of sense (S-), or antisense (AS-) Ndk) at an MOI of 20∶1. At 4 h post-phagocytosis, cells were fixed with 2.5% paraformaldehyde and mounted onto slides for confocal analysis. Bright field and merged fluorescent images are shown with an outline of the cell boundaries. Green signal indicates BCG that are not colocalized with dextran, while yellow signal shows colocalization of BCG with lysosomes. **C.** Quantification of data shown in panel B.

To assess directly the contribution of Ndk to mycobacterial virulence in the context of phagosome maturation arrest, we created a BCG strain with knocked-down expression of Ndk and examined its fate in RAW macrophages. Thus, mycobacterial shuttle vector pMV261 was engineered to express the Ndk gene in sense (S-Ndk) and anti-sense (AS-Ndk) directions in BCG. This resulted in a recombinant strain that expresses high level of Ndk antisense mRNA leading to abolition of Ndk protein expression as shown by western blot analysis (**Fig. 5A**). Additionally, compared to its parental strain, the Ndk knocked-down BCG strain showed no differences in its *in vitro* growth in culture media (**Fig. 5B**). To examine the contribution of Ndk to mycobacterial survival within the host, we infected RAW macrophages with wild-type BCG, or BCG expressing sense or antisense Ndk. Cells were then lysed and serial dilutions of recovered bacteria were plated on agar media plates at 24 h and 48 h post infection. The CFU counts (**Fig. 6A**) showed significantly decreased intracellular survival of BCG AS-Ndk strain. Specifically, at the 48 h time point, we observed a 1.5 Log decrease of BCG AS-Ndk CFUs relative to CFUs obtained from macrophages infected with wild-type and S-Ndk strains. Both control strains showed comparable survival rates.

The reduction of BCG survival by inhibiting Ndk expression in the macrophage further strengthens our findings that Ndk functions within host cells to inhibit phagolysosome fusion. Indeed, confocal analyses of macrophages loaded with FITC-DXT and infected with BCG strains showed a substantial number (∼40%) of BCG AS-Ndk phagosomes that fuse with lysosomes, whereas virtually no phagosome containing wild-type BCG showed interaction with the lysosomal compartments (**Fig. 6B and 6C**). Taken together, these data demonstrate that Ndk contributes significantly to successful long-term survival of pathogenic mycobacteria within the macrophage.

## Discussion

Earlier observations that arrest of phagosome maturation occurs only in macrophage ingesting live mycobacteria [40] suggested a mechanism dependent on active secretion of virulence factors capable of crossing the phagosomal membrane and deactivating critical regulators of phagosome biogenesis. Ndk (∼15 kDa) is one of many secreted mycobacterial proteins [28], [29] and the present study examined its effects on the regulation of phagosome biogenesis in the context of macrophage infection with mycobacteria.

Our hypothesis that Ndk acts as a potential inhibitor of phagosome maturation was supported by i) our recent findings that live mycobacteria express a GAP-like activity on Rab7 that has been recruited to the phagosome [17] and ii) by concomitant demonstration that mycobacterial Ndk acts as a GAP for Rho-GTPases [26]. Furthermore, several pathogens were shown to secrete proteins that act as GAP and facilitate their pathogenesis. For instance, *Pseudomonas aeruginosa* ExoS cytotoxin disrupts the actin cytoskeleton by acting as GAP for Rho-GTPases [41] and *Yersinia pseudotuberculosis* cytotoxic factor, YopE, depolymerizes the actin stress fiber through its GAP activity for Rho-GTPases [42]. Similarly *Legionella pneumophila* virulence factor LepB exhibits GAP activity towards host cell Rab1 GTPase to disrupt proper membrane cycling and activation [43].

The current study used purified recombinant Ndk adsorbed on latex bead in order to mimic intraphagosomal expression of proteins occurring during mycobacterial infection. In fact, a major advance in phagosome biology was made possible by using the latex bead system for analyses of many phagosome functions [44] and the option of coating these beads has been successfully used for examining modulation of phagosome biogenesis by several bacterial products [45], [46]. Thus, we have demonstrated that latex bead-mediated intracellular delivery of Ndk blocks phagosome fusion with FITC-DXT-loaded lysosomes as a result of exclusion of the Rab7 downstream effector RILP from the phagosomal membrane. These findings corroborate our observation of direct binding of Ndk to Rab7 *in vitro* and the subsequent dephosphorylation of the γ-phosphate of Rab7-GTP leading to inactive GDP-bound molecules.

The earlier observation that mycobacteria arrest phagosome maturation despite the presence of constitutively active mutant Rab7Q67L [16] suggested that Rab7 GTPase is not the only key regulator of phagosome maturation. In fact, membrane recruitment of another small GTPase, Rab5 was found to mediate EEA1-dependent phagosome fusion with early endosomes [13]. EEA1 is recruited to phagosomal membrane in the presence of the hVPS34 product PI3P and active Rab5 (GTP bound form) [30]. Binding of the EEA1 FYVE domain to PI3P stabilizes the interaction between Rab5 and EEA1 [47]. As EEA1 was shown to be excluded from *Mtb* and BCG phagosomes by a mechanism dependent on mycobacterial lipid phosphatase SapM [22], we examined an alternate mechanism mediated by mycobacterial GAP activity towards Rab5 GTPase. This hypothesis was confirmed by the demonstration of direct binding of Ndk to Rab5-GTP and its dephosphorylation, consistent with the observation of reduced recruitment of the Rab5 interacting effector EEA1 to phagosomes containing Ndk-coated latex beads. Thus, while SapM decreases EEA1 recruitment through hydrolysis of phagosomal PI3P [22], Ndk is acting through Rab5-GTP deactivation and attenuation of its interaction with hVPS34 leading ultimately to diminished phosphorylation of PI on the phagosomal membrane. This conclusion is consistent with previous findings that hVPS34 catalytic activity begins after binding to GTP-bound Rab5 [37].

**Figure 7:**
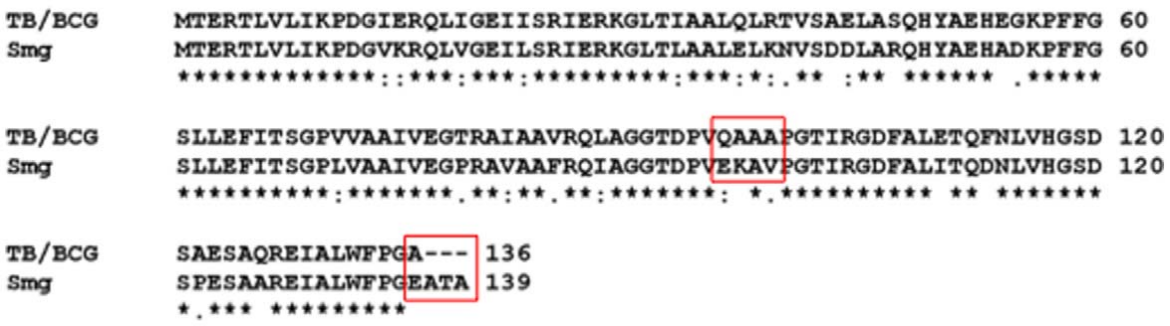
Sequence alignment of *Mtb*, BCG, and *M. smegmatis*. There is 100% homology between *Mtb* and BCG Ndk, which decreases to ∼80% when compared to *M. smegmatis* Ndk. Residues of difference that could potentially be important in the catalytic GAP activity are highlighted.

Of particular note is the finding that recombinant Ndk from *M. smegmatis* has a minor effect on Rab5 and no effect on Rab7 GTPase, consistent with its failure to block fusion of bead-containing phagosomes with lysosomes. These observations are in agreement with earlier reports showing that *M. smegmatis* fails to block phagosome maturation and are unable to ensure successful long term survival within the macrophage [21]. Comparison of amino acid sequence (**Fig. 7**) showed high homology (∼80%) between *Mtb*/BCG and *M. smegmatis* Ndks, including the conservation of the key catalytic histidine 117 (H117). However, the finding that mutation of the H117 residue does not affect GAP activity, despite significantly reduced functions in autophosphorylation, ATPase and GTPase activities [26], [28] suggest a specific domain –present in *Mtb* and BCG Ndks, but absent from that of *M. smegmatis*– involved in GAP catalytic activity, yet to be identified. Based on the crystal structure of *Mtb* Ndk, there is a distinct difference near the C-terminal region of the protein compared to that of *M. smegmatis* [48]. In particular, alanine 136, which is responsible for closing the polypeptide chain on itself with a salt bridge to arginine 4, is substituted with gluatmic acid in *M. smegmatis* Ndk. Furthermore, residues 95–99 show dissimilarities between the two proteins, and this is of importance due to being an integral part of the conserved ‘Kpn’ loop. Both of these differences suggest that the two Ndks differ in folding patterns, subunit stability and quaternary structure in ways that might affect functional activities of Ndk from *M. smegmatis*.

The ability to construct mutant strains of mycobacteria and test individual gene products for specific functions has significantly advanced discovery of virulence factors and our understanding of TB pathogenesis. To demonstrate the relevance of Ndk-mediated deactivation of Rab5 and Rab7 to the virulence of mycobacteria, we knocked-down Ndk gene expression in BCG using established antisense strategy [49], [50]. We observed increased fusion of phagosomes containing BCG AS-Ndk with lysosomes along with a significant decrease in bacterial survival within the macrophage. Thus Ndk appears to contribute to bacterial survival at early stages of infection. It is likely that mycobacteria continue to export more Ndk for alternate activities beyond the vicinity of the phagosome. Indeed, *Mtb* Ndk was shown to localize into the nucleus of the host cell and cause superoxide radical-mediated DNA cleavage [51]. Taken together, these findings suggest that Ndk contributes to the survival of *Mtb* at least by two consecutive events: i) arrest of phagosome maturation and subsequent establishment of the infection and ii) interference with host gene expression via DNAse activity.

Our finding that attenuated BCG AS-Ndk bacteria show normal growth is highly relevant to development of live TB vaccine. Thus far, two basic strategies are being employed in the development of novel live mycobacterial vaccines. The first strategy is to improve the immunogenicity of the existing BCG vaccine [52], [53] and the second is to use attenuated *Mtb* itself in order to mimic natural infection [54], [55]. Our demonstration of decreased survival of BCG AS-Ndk and promotion of phagolysosome fusion provides a rational and straightforward basis for extension to *Mtb* attenuation. By combining BCG AS-Ndk with additional mutations that disable virulence-promoting functions but preserve normal growth in culture media, it may be possible to develop a new generation of safe and effective attenuated *Mtb* vaccine strains that will have greater protective efficacy than BCG.

In summary, our findings suggest that mycobacterial Ndk possesses GAP activity that is trafficking within the host cell beyond phagosomes leading to inhibition of phagosome biogenesis processes dependent on Rab5 and Rab7. In doing so, Ndk contributes to intracellular survival and subsequent establishment of mycobacterial infection.

## Materials and Methods

### Reagents and Chemicals

Endotoxin-free culture reagents were from StemCell Technologies (Vancouver, British Columbia, Canada). Protease inhibitor mixture (PMSF, trypsin-EDTA) were purchased from Sigma-Aldrich (St. Louis, MO). Protein A-agarose beads were from Bio-Rad laboratories (Hercules, CA). Fetal calf serum (FCS), OPTI-MEM and HBSS were purchased from Gibco Laboratories (Burlington, Ontario). Lipofectamine 2000, Texas Red and fluorscein dextran (10,000 MW) were obtained from Invitrogen (Burlington, Ontario). TALON polyhistidine-Tag purification resin was purchased from Clontech (Mountain View, CA). Aldehyde/sulfate latex beads (diameter, 4 µm) were obtained from Interfacial Dynamics (Portland, OR). [γ-^32^P]-Guanosine 5′-triphosphate, was purchased from Perkin Elmer (Boston, MA).

### Antibodies

Rabbit anti-Rab5 and rabbit anti-Rab7 antibodies were purchased from Sigma-Aldrich. Rabbit anti-RILP antibody was described previously [39]. Secondary antibodies were purchased from Caltag Laboratories (Burlingame, CA). Monoclonal anti-rabbit IgG, native-peroxidase was purchased from Sigma-Aldrich. Mouse anti-Ndk antibodies were generated by injection of full-length recombinant *Mtb* Ndk (100 µg) solubilized in 150 µl Imject Alum (Pierce, Rockford, IL) adjuvant in FVB mice. Thereafter, the mice were boosted twice with 50 µg Ndk-Alum mixture after intervals of 14 days. Ten days after final injection, cardiac puncture was performed, and the titer of Ndk antiserum was determined by ELISA. The animal husbandry and immunization protocol were approved by the Animal Care Office at the University of British Columbia (Certificate number: A08-0873).

### Bacteria

*M. bovis* BCG (Pasteur 1173P2) was grown in Middlebrook 7H9 broth (Difco) supplemented with 10% (v/v) OADC (oleic acid, albumin and dextrose solution; Difco) and 0.05% (v/v) Tween 80 (Sigma-Aldrich) at 37°C on a rotating platform (50 rpm). For macrophage infection, bacteria in mid-log phase were harvested by 5 min centrifugation at 12,000 rpm. Bacteria were stained by FITC (Sigma) at a final concentration of 10 µg/ml at 37°C for 1 h. They were subsequently washed three times with 7H9 plus 0.05% tween and passed through 25 gauge needles several times prior to infection. Mycobacterial lysates were prepared by resuspending cell pellets in 350 µl of 50 mM Tris, 5 mM EDTA, 0.6% SDS, 0.05% NaN_3_, 1 mM PMSF. The cells were then mixed with 100 mg of glass beads and shaked in a bead beater for 15 second intervals for 10 times. Thereafter, lysates were separated from insoluble fractions and cell debris by centrifugation at 12,000 rpm at 4°C for 30 min.

### Plasmid Constructs

*Mtb* Rv2445c (*ndk*) was amplified from genomic DNA using the forward primer, TTG GGC CAT ATG ACC GAA CGG ACT CTG, containing an NdeI site, and reverse primer CAC CCG AAG CTT GGC GCC GGG AAA CCA, containing a HindIII site. *M. smegmatis ndk* was amplified from its genomic DNA using the forward primer TTG GGC CAT ATG ACT GAG CGG ACC CTC and the reverse primer GAA TTG AAG CTT GGC GGT GGC CTC GCC GGG, containing a NdeI site and HindIII site, respectively. The amplified genes were inserted into pET22b vector using the same restriction sites to generate a C-terminal his-tag fusion protein. Human *rilp* gene was amplified from pGEX-4T3-RILP [39], using the forward primer TTT CAT ATG GAG CCC AGG AGG GCG GCG, containing a NdeI site, and the reverse primer TTT AAG CTT GGC CTC TGG GGC GGC TGA, containing a HindIII site. The amplified insert was cloned into pET22b for His-tag expression. All plasmid constructs were subsequently verified by sequencing (Macrogen Co, South Korea). Plasmid vector expressing Rab7 and Rab5a in his-tag expression vector pET16b were previously described [56], [57]. Plasmids were transformed into *E. coli* strain BL21 and grown to an OD_600_ of 0.8 at 37°C and expression was induced with 0.2 mM IPTG at 22°C overnight. After centrifugation, bacteria were resuspended in PBS containing 1 mM PMSF, 1 mg/ml lysozyme for 30 min and lysed by sonication. Bacterial lysates were clarified by high-speed centrifugation and then purified on TALON polyhistidine-Tag purification resin. Fusion proteins were eluted in 250 mM immidazole. Rab7-GFP, Rab5-GFP plasmids were generated as previously described [58], [59]. RILP-DsRed plasmid was provided by Dr. Brett Finlay. GFP-EEA1 and GFP-2xFYVE were generated previously [60]. Ndk-DsRed plasmid was generated by inserting PCR-amplified Ndk between the XhoI and HindIII sites of pDsRed2-N1 (Clontech).

### Coating of Latex Microspheres with Proteins

Latex beads were coated with proteins as described previously [21], [23]. In brief, 10^8^ beads were washed twice with 25 mM MES buffer (pH 5.8) and resuspended in 500 µl of the same buffer containing 250 µg/ml of protein. After overnight incubation at room temperature on a shaker, latex beads were washed 3 times with PBS and resuspended in 1 ml of PBS containing 1% BSA. Based on the difference between protein concentration of the coating solution before and after incubation with beads, the coating was estimated to be 0.2–0.3 pg protein per bead.

To generate red fluorescent beads, BSA-beads were labelled with the PKH26 red Fluorescence linker kit (Sigma). In brief, beads were incubated in 1∶500 PKH dilution for 10 min at 37°C. Beads were then washed three times and resuspended in PBS.

### Cell Culture, Transfection and Infection

RAW 264.7 (American Type Culture Collection, Manassas, VA) were maintained in 10 cm diameter culture dishes (Corning Inc., Corning, NY) at a density of ∼10^5^ per cm^2^ in DMEM containing 5% FCS and 1% each of L-glutamine, HEPES, non-essential amino acids (100× solution, StemCell). Prior to transfection, RAW cells were washed extensively and harvested by scraping. Approximately 5×10^5^ cells were allowed to adhere on coverslips in 24-well plates. Cells were then transfected with the GFP constructs described above using Lipofectamine 2000 (Invitrogen) according to the manufacturer's instructions. Twenty-four hour post-transfection, cells were washed and infected with latex beads or mycobacteria at multiplicity of infection (MOI) 2∶1 or 20∶1, respectively. Post-infection samples were washed extensively and partially attached and non-ingested beads/bacteria are removed by trypsin treatment followed by multiple washes.

### Fluorescence Microscopy

Coverslips were mounted on microscope slides in FluorSaveTM (Calbiochem-Novabiochem, La Jolla, CA) to minimize photobleaching. Slides were then examined by digital confocal microscopy using an Axioplan II epifluorescence microscope (Carl Zeiss Inc., Thornwood, NY) equipped with 63×/1.4 Plan-Apochromat objective (Carl Zeiss Inc). Images were recorded using a CCD digital camera (Retiga EX, QImaging, Burnaby, BC, Canada) coupled to the Northern Eclipse software (Empix Imaging, Inc., Mississauga, ON, Canada).

### GAP (GTPase Activating Protein) Activity Assay

Rab5 or Rab7 (1 µg) were loaded with [γ-^32^P]GTP by incubation with 10 µCi of [γ-^32^P]GTP in 100 µl of reaction buffer (50 mM HEPES pH 7.4, 50 mM NaCl, 0.1 mM DTT, 5 mM EDTA and 1 mg/ml BSA) for 10 min at 37°C. 10 mM MgCl_2_ was then added (to terminate the reaction) and incubated on ice for 10 min. Thereafter, 5 µl of each reaction mixture were spotted onto nitrocellulose membrane. Unbound material was removed with extensive washing with cold wash buffer (25 mM HEPES pH 7.4, 50 mM NaCl, 1 mM MgCl_2_, 1 mM DTT) and membrane squares were then incubated in the presence or absence of 10 µg/ml *Mtb* or *M. smegmatis* Ndk for 2 h at room temperature. After 3 washes with cold wash buffer, membrane-associated radioactivity was determined by autoradiography and quantification was done by measuring membrane counts in a scintillation counter.

### Antisense Knock-Down

*Mtb ndk* gene was PCR amplified using the forward primer CCG AAG CTT GTG ACC GAA CGG ACT CTG GTA, and reverse primer CCG AAG CTT CTA GGC GCC GGG AAA CCA GAG, both using the restriction site HindIII. The 411bp insert was cloned into pMV261 cut at HindIII of the multicloning site. Prior to ligation, the pMV261 was treated with CIAP to remove the 5′ phosphate and prevent self-ligation. Positive clones were then digestion checked by PvuII. Clones with *ndk* inserted in the sense orientation would give a fragment of 4800 bp and 60 bp, while clones of *ndk* inserted in the anti-sense orientation would give fragment sizes of 4400 bp and 360 bp. The plasmids were then electroporated into competent BCG and plated on 7H10 media supplemented with OADC and containing 25 µg/ml kanamycin.
